# Pharyngeal Residue Scoring in Fiberoptic Endoscopic Evaluation of Swallowing: Reliability Comparison and Applicability Among Different Scales

**DOI:** 10.1007/s00455-024-10669-3

**Published:** 2024-02-08

**Authors:** Federica Messina, Sara Rocca, Beatrice Manca, Letizia Scarponi, Aurora Ninfa, Antonio Schindler, Nicole Pizzorni

**Affiliations:** Department of Biomedical and Clinical Sciences, Università Degli Studi Di Milano, 20157 Milan, Italy

**Keywords:** Deglutition disorders, Pharyngeal residues, Fiberoptic endoscopic evaluation of swallowing, Reliability

## Abstract

Several scales to assess pharyngeal residue in Fiberoptic Endoscopic Evaluation of Swallowing (FEES) are currently available. The study aimed to compare the reliability and the applicability in real clinical practice among four rating scales: the Pooling Score (P-SCORE), the Boston Residue and Clearance Scale (BRACS), the Yale Pharyngeal Residue Severity Rating Scale (YPRSRS), and the Residue Ordinal Rating Scale (RORS). Twenty-five FEES videos were evaluated four times, once for each scale, by four speech and language pathologists. To test intra-rater reliability, the same raters re-assessed the videos two weeks apart. To test the applicability, raters recorded the time required to complete each assessment and the perceived difficulty/ease on a visual-analog scale (VAS). The intra-rater and the inter-rater reliability were calculated with Cohen’s weighted Kappa and the Fleiss weighted Kappa, respectively. Time and perceived difficulty/ease scores were compared. The intra-rater reliability analysis showed almost perfect agreement for YPRSRS (*k* = 0.91) and RORS (*k* = 0.83) and substantial agreement for P-SCORE (*k* = 0.76) and BRACS (*k* = 0.74). Pairwise comparison showed no significant differences among the scales. The inter-rater reliability for the YPRSRS (*k* = 0.78) was significantly higher than P-SCORE (*k* = 0.52, *p* < 0.001), BRACS (*k* = 0.56, *p* < 0.001), and RORS (*k* = 0.65, *p* = 0.005). The BRACS required the longest time (*p* < 0.001) and was perceived as the most difficult scale (*p* < 0.001). The RORS was perceived as the easiest scale (*p* < 0.05). In conclusion, the YPRSRS showed the highest reliability, while raters perceived the RORS as the easiest to score. These results will allow clinicians to consciously choose which scale to use in clinical practice.

## Introduction

The assessment of dysphagia involves an examination of two constructs: the efficacy of swallowing, which is the ability to ingest all the substances needed to remain nourished and hydrated, and the safety of swallowing, which is the ability to ingest all needed substances without any respiratory complications [1]. Pharyngeal residue is a sign of impaired efficacy [2]. Incomplete bolus clearance may be caused by an impairment of oropharyngeal propulsion or upper esophageal sphincter relaxation and opening [3]. Furthermore, residue is associated with an increased risk of penetration and aspiration after swallowing [4–6].

Fiberoptic Endoscopic Evaluation of Swallowing (FEES) and Videofluoroscopy are considered the gold standards for the assessment of dysphagia and for the detection of pharyngeal residue [7]. During FEES, the complete inspection of the velopharynx, oropharynx, pharynx, and larynx is possible and, after the swallow, the direct presence of bolus residue can be observed [8]. In addition, FEES has found to be more sensitive than videofluoroscopy to detect pharyngeal residue [9, 10].

Different scales for the evaluation of pharyngeal residue on FEES are currently available. They can be generally defined as binary (presence/absence of residue), ordinal (to differentiate increasing amounts of residue), or estimation (to estimate the amount of residue compared to the initial bolus) [11]; currently, there are no studies comparing reliability and clinical applicability of different residue scales.

The Yale Pharyngeal Residue Severity Rating Scale (YPRSRS) [12] is an image-based ordinal rating scale, which allows to assess the amount of residue in the valleculae and the pyriform sinus. A systematic review [11] of pharyngeal residue severity scales based on FEES reported the YPRSRS [12] as the most valid, reliable, and generalizable scale among those included in the review [13–18]. Despite this, a limitation of the YPRSRS is the difficulty scoring unilateral residue compared to bilateral residue [19]. A previous study showed that the reliability of the YPRSRS based on video was poorer compared to previous data on reliability of the scale gathered on images [20]. Moreover, the bolus consistency influenced the scale’s construct validity; indeed, the degree of construct validity was greater in pureed and solids food than in liquids [20].

Another scale for the evaluation of pharyngeal residue is the Boston Residue and Clearance Scale (BRACS), for which the original publication reported high inter-rater and test–retest reliability, concurrent validity, and internal consistency [21]. The BRACS is an 11-point ordinal rating scale to assess the residue’s amount and location, the presence, and effectiveness of spontaneous clearing swallows [21]. A systematic review of the psychometric properties of visuoperceptual measures of fiber-endoscopic evaluations of swallowing reported the BRACS to have limited positive evidence for reliability and moderate positive evidence for structural validity [22]. The BRACS also received indeterminate scores for internal consistency, content validity, and hypothesis testing categories in the same review. Although the scale had sufficient evidence for some psychometric properties, the overall psychometric quality and the quality of all measures retrieved were relatively weak [22]. Despite being a highly detailed scale, an instruction manual is not currently available [19], and the scale was only published in the initial validation study.

The Pooling-score (P-Score) is an ordinal rating scale to assess site, amount, and management of residue. The scale showed a very high correlation among the scores attributed by different raters and seemed unaffected by the bolus consistencies [16]. The critical points of the scale are the lack of severity definitions and anatomical landmarks [19].

A study about the observers’ agreement on FEES measurements reported another visuoperceptual scale, the Residue Ordinal Rating Scale (RORS), for which the observers’ agreement was influenced by the bolus consistency and not by the dysphagia etiology [23]. The RORS provides two separate scores for residue amount in vallecula and in pyriform sinuses.

Usually, visuoperceptual scales are used by the clinicians performing the FEES in real time during the instrumental assessment of patients with dysphagia. In many European countries FEES is performed by phoniatricians and otorhinolaryngologist, while in Anglo-American countries it is performed by SLPs too [24]. Applying the scales to each assessment can help clinicians monitor the evolution of the patient’s swallowing or response to treatment over time. The visuoperceptual nature of the previous scales inevitably leads to a subjective interpretation. Thus, further investigations are needed to understand reliability of the different existing scales [23]. Furthermore, the reliability of different scales available to assess residue on FEES has not been compared to date.

The study’s objectives were to compare the intra- and inter-rater reliability of four different scales for assessing pharyngeal residue on FEES and to compare the perceived difficulty/ease and the time required to assign the score among the scales. A good reliability is expected for all scales. Furthermore, the results may provide information for clinicians to choose which scale to apply in clinical practice and for research purposes, considering the reliability and the applicability.

## Material and Methods

The study has an observational, prospective, and cross-sectional design. The project was carried out following the Declaration of Helsinki of the World Medical Association (WHO) and was approved by the Ethical Committee of the University of Milan (approval n. 102/20). All data were processed in a pseudonymized form.

### Participants

The raters were four speech and language pathologists (SLPs) with 0 to 5 years of experience in dysphagia (mean 3 ± 2.16 years). Specifically, one rater just graduated with the Bachelor’s Degree in SLP, two raters had a Master Degree in Rehabilitation Sciences and were involved in several research projects in the field of dysphagia as PhD candidate or research assistant at the time of the assessment, while the remaining SLP was working as clinician with patients with dysphagia and attended a Master in Dysphagia. All raters attended a 3-h training on the application of pharyngeal residue assessment scales. The training was conducted by a SLP with ten years of experience in dysphagia and FEES interpretation. It included a theoretical explanation of the scales and a practical application on fifteen videos with a discussion among the participants of the scores assigned to each scale. The videos used in training were not included in the study. No learning test was conducted at the end of the training.

### Pharyngeal Residue Assessment Scales

Four different residue assessment scales were used. The four scales were selected from previous reviews [11, 22] on FEES metrics. Specifically, we selected the scales developed to evaluate pharyngeal residue of foods and liquids and indicated for clinical purposes.

The YPRSRS [12] is an ordinal rating scale. Two different scores for residue in the valleculae and pyriform sinus are provided. In both cases, the score ranges from 1 (none) to 5 (severe). For each level, identifying the amount of residue is facilitated by a percentage range, an operational description, and an anchor image. The scale was initially validated on FEES frames but has recently been shown to be valid and reliable also in FEES videos [20].

The BRACS [21] is an ordinal rating scale. The final score ranges from 0 to 10. The higher the score, the more severe the presence of residue. The score is obtained by summing five different subscores:

1. The location and amount of residue. For every location (lateral pharyngeal wall/posterior pharyngeal wall, the base of the tongue, valleculae/tip of epiglottis, left lateral channel/left pyriform recess, right lateral channel/right pyriform recess, post-cricoid region, left arytenoid/left aryepiglottic fold, right arytenoid/right aryepiglottic fold, inter-arytenoid space, laryngeal surface of the epiglottis; the laryngeal surface of aryepiglottic fold/false vocal folds, anterior commissure/true vocal folds/posterior commissure), the amount of residue is reported (none/coating, mild, moderate, severe). The worst score obtained from any location is considered the subscore.
2. The presence of residue in 4 or more locations.
3. The presence of residue in the vestibule.
4. The presence of spontaneous clearing swallows.
5. The effectiveness of spontaneous or clued clearing swallows by the third swallow (80–100% cleared; 20–80% cleared; 0–20% cleared).

The P-Score [16] is an ordinal rating scale. The final score ranges from 4 (no dysphagia) to 11 (severe dysphagia) and is obtained by adding three different subscores:

1. The site of residue (valleculae, marginal zone, pyriform sinus, vestibule/vocal cords, lower vocal cords). The worst location is considered if the residue is present in more than one location.
2. The amount of residue (coating, minimum, maximum). If residue is present in more than one location, the amount is evaluated in the worst location.
3. The management of residue (< 2 dry swallows, 2–5 dry swallows, > 5 dry swallows). If residue is present in more than one location, the management is evaluated in the worst location.

The RORS [23] is an ordinal rating scale. Two different scores for residue in the valleculae and pyriform sinus are provided. In both cases, the score ranges from 0 (no pooling) to 2 (filling more than 50% of the valleculae/severe pooling up to complete sinus filling).

### FEES Videos

The videos were selected from databases of Luigi Sacco University Hospital in Milan and Clinical Scientific Institutes Maugeri in Milan. The recordings were previously collected for research purposes and were randomly selected and anonymized. At Luigi Sacco University Hospital in Milan, a XION EF-N flexible endoscope with a diameter of 3.4 mm and length of 320 mm (XION GmbH, Berlin, Germany) was used. It was connected to a MATRIX LED DUO light source and supported by the ENDOSTROB E video processor (XION GmbH, Berlin, Germany). The recordings have been stored by the DAISY VIEWER 2.0 software (INVENTI S.r.l., Padova, Italia). At Clinical Scientific Institutes Maugeri of Milan, a PENTAX FNL-10RBS portable, flexible endoscope with a diameter of 3.5 mm and length of 300 mm (Pentax Europe GmbH, Hamburg, Germany) was used. It was supported by the PENTAX EPK-1000 video processor (PENTAX Europe GmbH, Hamburg, Germany).

Regarding the consistency definition, the terminology of the International Dysphagia Diet Standardization Initiative (IDDSI) framework was used [25]. The evaluation was performed with thin liquids (5–10–20 mL of blue-dyed water room temperature × 3 trials for each volume; IDDSI 0; < 50 mPa·s at 50 s^−1^ and 300 s^−1^), pureed food (5–10–20 mL of Crème Line Valilla Nutrisens—Nutrisens Italia SRL, Turin, Italy—pudding × 3 trials for each volume; IDDSI 4; 2583.3 ± 10.41 mPa·s at 50 s^−1^ and 697.87 ± 7.84 mPa·s at 300 s^−1^), and regular food (half 8 g of Frollini Monviso—Monviso group SRL, Andezeno, TO, Italy—biscuit × 2 trials; IDDSI 7 Regular). The Haake Viscotester 550 (Thermo Electron GmbH, Dieselstr, Germany) was used for the viscosity analyses; viscosities below 300 mPa·s were performed with the system MV1 (gap:0.96 mm) and viscosities over 300 mPa·s with the system SV1 (gap: 1.45 mm). The shear rate for the swallowing process can range from 1 to 1000 s^−1^ [26]. As in previous studies [27, 28], values of 50 and 300 s^−1^ were used to reflect viscosity at the oral or pharyngeal stage of swallowing. In order to evaluate specific consistencies and bolus volumes (three boluses of 10 ml for thin liquids and pureed food; two boluses of half biscuit for regular food), the videos were cut and reassembled. For each video, raters assigned a score of pharyngeal residue to each consistency. Videos of 25 subjects (11 men and 14 women, with a mean age of 57.48 ± 16.88) were included. Five healthy subjects (20%) and 20 subjects with neurological diseases were selected: 5 with Parkinson’s disease (20%), 5 with Huntington’s disease (20%), 5 with Amyotrophic Lateral Sclerosis (20%), and 5 with Myotonic Dystrophy type I (20%). The inclusion criteria for healthy subjects were age between 18 and74 years and a normal 3-oz Water Swallow Test [29]; the exclusion criteria were: known history of swallowing disorders, respiratory diseases, rheumatic diseases, metabolic disorders, gastroenterologist diseases, neurological diseases, hematologic diseases, voice disorders, and neoplasms. The inclusion criteria for the pathological subjects were age > 18 years and diagnosis of neurological disease; the exclusion criteria were known history of head and neck cancer and comorbidity with other neurological diseases. Both healthy and pathological subjects were included to represent different levels of dysphagia (Dysphagia Outcome and Severity Scale [30] 2–7, mean 4.76 ± 1.36) and pharyngeal residue severity based on characteristics phenotypes of different neurological diseases [31].

### Procedures

Twenty-five FEES videos were randomized and evaluated in two different assessment sessions, at least two weeks apart, one to each other. At each assessment session, the independent SLPs evaluated each FEES video four times in a random order for each residue assessment scale. Additionally, the order of the scales to apply to FEES videos were randomized among raters. Regarding the time to evaluate the amount and site of residue, raters were given the following guidance: “Assess the amount of residue at the end of the worst swallowing act, before any clearing swallows.” For the first assessment, the time was measured in minutes and seconds and was started from the beginning of the video up to the attribution of the score. The raters could stop the videos or play them multiple times. The raters also provided a score to the perceived difficulty/ease on a visual-analog scale (VAS) and the time required to complete the scales. The VAS ranged from 0 (extremely easy) to 10 (extremely difficult) on a 10 cm line. No middle anchor was provided.

### Statistical Analysis

A statistical analysis was performed with the IBM SPSS Statistics 27.0 package for Windows (SPSS Inc, Chicago, IL) and the Agreetest software (Kilem L. Gwet) [32].

The YPRSRS and the RORS provide two scores for valleculae and pyriform sinuses; the BRACS and the P-score provide a single score that considers all sites. Thus, the analysis was based on 600 ratings for the YPRSRS and the RORS (4 raters rated each of the 25 videos for valleculae and pyriform sinuses and separately for thin liquids, pureed food, and regular food) and 300 ratings for the BRACS and the P-score (4 raters rated 25 videos providing a separate score for thin liquids, pureed food, and regular food).

To determine the intra-rater reliability, the weighted Cohen’s Kappa (quadratic weighting) [33] was calculated for each rater; the Average Cohen’s kappas of distributions were compared among the scales using the one-way analysis of variance (ANOVA) with post hoc (Tukey’s Hsd) analysis.

To determine the inter-rater reliability, the Fleiss Kappa [34] was calculated for each scale; Fleiss Kappa values were compared using the unpaired *t-test*. Significance was set at *p* < 0.05.

Concerning Cohen’s Kappa statistics, the levels of agreement were determined according to the following criteria: Kappa values of 0 were considered to indicate poor agreement, 0.00–0.20 slight agreement, 0.21–0.40 fair agreement, 0.41–0.60 moderate agreement, 0.61–0.80 substantial agreement, 0.81–1.00 almost perfect agreement [33]. For the Fleiss Kappa, the following benchmark was adopted: < 0.40 poor, 0.40–0.75 intermediate to good, and > 0.75 excellent [34].

To analyze the perceived difficulty/ease and the time required to assign a score for pharyngeal residue among the scales, VAS (cm) and time (s) median (IQR) for each scale were calculated from the data of each rater. The Kolmogorov–Smirnov test assessed the normality of the continuous variables. As none of the continuous variables was normally distributed, pairwise comparison among non-normally distributed variables was made using the Kruskal–Wallis test and post hoc Dunn’s test. Significance was set at *p* < 0.05.

## Results

### Intra-rater Reliability

The values of Cohen’s Weighted Kappa are reported in Table 1, while pairwise comparison using the ANOVA with post-hoc analysis (Tukey’s Hsd) is reported in Table 2. The analysis showed almost perfect agreement for the YPRSRS and the RORS (0.81 < *k* < 1.00) and substantial agreement for the BRACS and the P-SCORE (0.61 < *k* < 0.80). Averaged Cohen’s Weighted Kappa of the YPRSRS was higher than other scales, while the lowest values were found for the BRACS. However, in the pairwise comparison, there were no significant differences among the intra-rater reliability of all scales (F (3, 12) = 2.46; *p* = 0.113).

**Table 1:** Intra-rater reliability among all raters and for each individual rater

	Intra-rater across all raters (n = 4)	Intra-rater R1	Intra-rater R2	Intra-rater R3	Intra-rater R4
	Averaged Cohen’s Weighted Kappa ± Se	Cohen’s Weighted Kappa ± Se	Cohen’s Weighted Kappa ± Se	Cohen’s Weighted Kappa ± Se	Cohen’s Weighted Kappa ± Se
YPRSRS^a^	0.91 ± 0.03	0.95 ± 0.01	0.91 ± 0.02	0.83 ± 0.04	0.96 ± 0.01
BRACS^b^	0.74 ± 0.07	0.73 ± 0.05	0.58 ± 0.07	0.71 ± 0.06	0.93 ± 0.03
P-SCORE^b^	0.76 ± 0.05	0.82 ± 0.05	0.67 ± 0.08	0.68 ± 0.08	0.88 ± 0.03
RORS^a^	0.83 ± 0.04	0.88 ± 0.03	0.77 ± 0.04	0.76 ± 0.04	0.89 ± 0.03

Note: ^a^based on 600 ratings; ^b^based on 300 ratings

**Table 2:** Pairwise comparison for the intra-rater reliability across all raters

	Intra-rater
	One-way analysis of variance (ANOVA)
	*p*-Value
YPRSRS vs P-SCORE	.209
YPRSRS vs BRACS	.111
YPRSRS vs RORS	.643
P-SCORE vs RORS	.803
P-SCORE vs BRACS	.977
BRACS vs RORS	.576

Note: pairwise comparison adjusted with Tukey HSD method

### Inter-rater Reliability

The values of Fleiss Kappa are reported in Table 3, while pairwise comparison using the *t*-test is reported in Table 4. The analysis showed excellent values for the YPRSRS (*k* > 0.75) and intermediate to good values for the other scales (0.40 < *k* < 0.75). Fleiss Kappa of the YPRSRS was significantly higher than other scales, while lowest values were found for the P-SCORE.

**Table 3:** Inter-rater reliability

	Inter-rater
	Fleiss K ± se
YPRSRS^a^	0.78 ± 0.03
BRACS^b^	0.56 ± 0.05
P-SCORE^b^	0.52 ± 0.06
RORS^a^	0.65 ± 0.04

Note: ^a^based on 600 ratings; ^b^based on 300 ratings

**Table 4:** Pairwise comparison for the inter-rater reliability obtained with the unpaired *t*-test

	Inter-rater	
	*t*-test	
	*t*_(df)_	*p*-Value
YPRSRS vs P-SCORE	*t*_(104.6)_ = 3.88	** < .001**
YPRSRS vs BRACS	*t*_(170.9)_ = 0.91	** < .001**
YPRSRS vs RORS	*t*_(277.8)_ = 2.85	**.005**
P-SCORE vs RORS	*t*_(126.1)_ = 1.82	.071
P-SCORE vs BRACS	*t*_(142.1)_ = 0.47	.640
BRACS vs RORS	*t*_(145.6)_ = 1.46	.148

Note: Significant p-values are reported in bold

### Time and Perceived Difficulty/Ease

The values of time and the perceived difficulty/ease median (IQR) are reported in Table 5, while pairwise comparison using the Kruskal–Wallis test with post-hoc analysis (Dunn’s test) is reported in Table 6. Regarding the scoring time, the BRACS took significantly longer to score than other scales. The RORS required less time to be scored, even though no significant differences were found (H (3) = 84.95, *p* < 0.001). Regarding the perceived difficulty/ease, the RORS was found significantly easier to score than other scales, while the BRACS showed a significantly higher difficulty.

**Table 5:** Time and perceived difficulty/ease median values across the 4 raters

	Time (s)—median (IQR)	Perceived difficulty/ease (cm)—median (IQR)
YPRSRS	6:09 (5:05–7:17)	3.7 (2.3–5.18)
BRACS	9:45 (7:01–12-12)	5.85 (4.8–7.00)
P-SCORE	6:00 (4:40–7:54)	3.65 (2.63–5.08)
RORS	5:51 (4:30–8:00)	2.9 (1.93–4.48)

**Table 6:** Pairwise comparison of time and perceived difficulty/ease obtained using the Kruskal–Wallis test with post-hoc analysis (Dunn’s test)

Pairwise comparison	*p* Value	
	Time	Perceived difficulty/ease
YPRSRS vs P-SCORE	.464	.622
YPRSRS vs BRACS	** < .001**	** < .001**
YPRSRS vs RORS	.799	**.043**
P-SCORE vs RORS	.323	**.012**
P-SCORE vs BRACS	** < .001**	** < .001**
RORS vs BRACS	** < .001**	** < .001**

Note. Significant p values are reported in bold

## Discussion

In this project, the intra- and inter-rater reliabilities of four scales for the assessment of pharyngeal residue during FEES were compared for the first time. The clinical applicability of the scales was also analyzed by comparing the difficulty and the time required to assign the score of each scale.

### Intra-rater Reliability

The intra-rater reliability analysis showed almost perfect agreement for the YPRSRS and the RORS and substantial agreement for the P-SCORE and the BRACS.

In the original publication of the YPRSRS, excellent intra-rater reliability for valleculae and pyriform sinus was found [12]. In another article about the YPRSRS and the effect of bolus consistency, comparable values were reported [20].

The original P-SCORE publication calculated intra- and inter-rater reliability using the intraclass correlation coefficient (ICC), both for the overall scale score and each subscore (the site, the amount, and the management of residue). The ICC values were excellent [16]. Even though different statistical analyses have been used and a direct comparison is not possible, the level of reliability seems to be different from this research in which substantial agreement for the P-SCORE was found. A direct comparison between the results of this study to those of previous articles is not possible for the BRACS and the RORS, for which intra-rater reliability was not provided.

In the pairwise comparison, no significant differences among the scales were found. Although all scales obtained good values of agreement, the intra-rater reliability of the YPRSRS and the RORS was higher than the P-SCORE and the BRACS. To explain this, the first hypothesis could be related to the simpler construction of the YPRSRS and the RORS that consider only the site (valleculae and pyriform sinus) and the amount of residue. In addition, these scales do not evaluate the management of residue, unlike the P-SCORE and the BRACS. In fact, the second hypothesis is that the management of residue represents a critical point for the intra-rater reliability.

### Inter-rater Reliability

The inter-rater reliability was excellent for the YPRSRS and intermediate to good for the BRACS, the P-SCORE, the RORS. As reported before, a systematic review on pharyngeal residue severity scales based on FEES reported the YPRSRS as the most valid, reliable, and generalizable scale [11]. Another systematic review about the psychometric properties of visuoperceptual measures of fiber-endoscopic evaluations of swallowing reported strong positive reliability for the YPRSRS [22]. Also, in the original publication of the YPRSRS, very good to excellent inter-rater reliability for valleculae and pyriform sinus was found [12]. In another work about the YPRSRS and the effect of bolus consistency, comparable values were reported [20]. The reliability of the YPRSRS that was established in these works seems to be confirmed. In the original publication of the BRACS, excellent inter-rater reliability was reported [21]. The higher values reported by the original study may be due to how the reliability was verified by recruiting a SLPs rater who worked at the same institution and participated in scale development. In the original publication of the P-SCORE, the ICC of intra and inter-rater reliability for the site, the amount, the management, and the P-SCORE total was found to be excellent [16]. As already reported, different statistics do not allow a direct comparison. Despite this for both BRACS and P-score, the level of reliability seems to be different from this work, in which intermediate to good inter-rater reliability for the BRACS and the P-SCORE was found. A direct comparison between the results of this study to those of previous articles is not possible for the RORS, for which inter-rater reliability is not provided.

In the pairwise comparison, the inter-rater reliability of the YPRSRS was significantly higher than other scales (*p* < 0.05). As previously reported, in the YPRSRS, the identification of the amount of residue is facilitated by a percentage range, an operation description, and an anchor image [12]. The possibility of relying on more than a single operational definition may have contributed to increased agreement among raters during the two assessment sessions. This result can be particularly relevant in clinical practice when the patient is evaluated by different professionals and for research purposes when different raters assess the same FEES recording.

### Time and Perceived Difficulty/Ease

The BRACS required the longest time (*p* < 0.001) and was perceived as the most difficult scale to score (*p* < 0.001). This could be because the BRACS is a very detailed scale and includes several different items to be scored. This result could make the scale less applicable in everyday clinical practice. Furthermore, clear instructions for using the scale have not been published. In addition, the study included video recordings that the raters could interrupt as often as they wanted. In a “live FEES examination” scenario, the application of this scale could be even more difficult. Despite this, the large number of items could be an advantage in the research field when a more precise assessment of pharyngeal residue is needed. The RORS was found to be the most accessible scale to score (*p* < 0.05); This could be related to the simple construction of the scale that considers only the site (valleculae and pyriform sinus) and the amount of residue. Although the YPRSRS evaluates the same variables, the RORS was still found to be easier. To explain this, it is possible that the raters found some difficulties in selecting a YPRSRS score based on the different severity rating definitions of the YPRSRS (the percentage range, the operation description, and the anchor image) that may not always be unambiguous.

## Limitations

There are several limitations in this study. The relatively low number of raters and the lack of a sample size calculation represent a first limitation. However, the length of the task (25 videos assessed for 8 times) was an obstacle in finding volunteer raters. Future studies should include a larger sample size. Second, the results were not analyzed considering the influence of years of experience in interpreting FEES evaluations, neither considering the etiology of dysphagia or comparing patients and healthy subjects. Timing variable was collected and self-reported by clinicians, so the authors cannot ensure the rigor of data collection. However, the authors trained the raters to be as accurate as possible in reporting the data.

FEES videos were executed using a flexible fiberscope, in future studies, the use of a video endoscope could lead to different results from those presented here. FEES were performed with blue-dyed water. The bolus opacity is known to influence residue visualization [35]; thus, another type of dye could have affected the results.

All scales included in the present study are based on categorical rating system. Recently, Curtis et al. developed the Visual Analysis of Swallowing Efficiency and Safety (VASES) [36], a new method to evaluate pharyngeal residue, penetration, and aspiration during FEES based on a VAS. The VASES was not included in the present study as it had not yet been published at the time of study design and data collection. Nevertheless, future studies should compare the performance of categorical scales and the VASES for the assessment of residue in FEES.

## Conclusions

All the investigated scales for the assessment of pharyngeal residue in FEES showed intermediate to good inter-rater and substantial intra-rater agreement. However, the YPRSRS had the highest reliability and average values of time and difficulty which make it suitable for both research and clinical practice. The BRACS was the longest and the most challenging scale, whereas the RORS was perceived as the easiest. The results of the present study provide clinicians with more information about scales to assess pharyngeal residue in FEES to guide them in selecting the most appropriate scale to use in their clinical practice, by balancing both the reliability and the applicability of each scale.

## Data Availability

The data that support the findings of this study are available from the corresponding author upon reasonable request.
